# Investigation of EDF evolution and charged particle transport in E × B plasma based negative ion sources using kinetic simulations

**DOI:** 10.1038/s41598-023-45656-5

**Published:** 2023-11-16

**Authors:** Miral Shah, Bhaskar Chaudhury, Mainak Bandyopadhyay

**Affiliations:** 1 Institute for Plasma Research, Gandhinagar, 382428 India; 2 Group in Computational Science and HPC, DA-IICT, Gandhinagar, 382007 India; 3 ITER-India, Institute for Plasma Research, Gandhinagar, 382428 India; 4 Homi Bhabha National Institute, Anushaktinagar, 400094 India

**Keywords:** Magnetically confined plasmas, Plasma-based accelerators

## Abstract

A spatially varying transverse magnetic filter field (TMF) is present in an E $\times$ B plasma-based negative ion source to improve negative ion yield. The TMF strength ranges from 1 to 10 mT, causing the plasma electrons to become magnetized while leaving the ions either unmagnetized or partially magnetized. As a consequence, plasma drift, particle trapping, double layer (DL), and instabilities are observed in a negative ion source. The transport of plasma through the TMF is influenced by these phenomena, subsequently affecting the energy distribution functions (EDFs) of both electrons and ions in the plasma. Measurement of EDFs in such systems is a challenging task due to the presence of a strong magnetic field. To address this, a 2D-3V Particle-in-Cell Monte Carlo Collision (PIC MCC) model is employed to study the spatio-temporal evolution of the EDFs separately for electrons and ions. The electron EDF (EEDF) remains Maxwellian, while ion EDF (IEDF) gradually transitions to non-Maxwellian as measurements are taken closer to the TMF region. The present study reveals that the IEDF is more sensitive to the operational conditions compared to the EEDF, as evidenced by the changes observed in both EDFs under different plasma operational conditions.

## Introduction

The study of plasma transport in partially magnetized E$\times$B plasma devices and the instabilities that arise from self-generated electric fields has indeed gained significant attention in recent times due to its wide range of applications^1–5^. These applications include negative ion sources for nuclear fusion, material processing, Hall thrusters, electron cyclotron resonance sources, ion-mass separators, and linear magnetized machines for basic studies. In particular, low-temperature plasma-based negative ion sources are of great significance in neutral beam injection systems, which play a crucial role in heating nuclear fusion reactors^6^. Magnetic field configuration in negative ion sources, particularly the transverse magnetic filter (TMF) field plays an important role not only to improve negative ion yield but also to control co-extracted electron current when negative ions are extracted as an ion beam by an electrostatic ion beam extractor system^7,8^. Plasma after being produced in the source region flows out towards the expansion region and then towards extraction region through the TMF field^9^. The spatially varying TMF field controls the plasma flow and cools down the electrons near extraction region, which helps to reduce the negative ion destruction and the co-extracted electron current during beam extraction. The self-consistent generation of gradients of plasma density, temperature, and space potential within the plasma volume is a consequence of the spatially varying TMF field. These gradients serve as a source of free-energy that can trigger instabilities^10,11^ and affect the plasma diffusion through TMF^12^.

Experimental and simulation studies on low temperature plasmas typically involve analyzing plasma parameters, including plasma density, electron temperature, and potential, as well as their respective profiles to understand plasma transport^13–19^. Energy distribution functions (EDFs) of the electrons and ions determine the plasma parameters, and various reaction rates of electron and ion induced processes that generate reactive species^20,21^. EDFs measurements are normally done by electrostatic probes but are challenging in such experiments due to the presence of magnetic field^20–26^. However, the Particle In Cell Monte Carlo Collisions (PIC-MCC) simulations facilitate the study of the evolution of EDFs. Plasma parameters such as mean electron energy, plasma conductivity, Debye length, and ion sound speed are calculated from the low energy part of EEDFs (electron EDFs). While inelastic collision rates and floating wall potential are calculated from the high energy part of EEDFs. IEDFs (ion EDFs) are associated with surface effects and ion dynamics plays an important role in the surface production route of negative ions^27,28^. While several studies on the EEDF have been reported, there is a noticeable lack of literature focused on IEDFs and their role in plasma transport through the TMF in negative ion sources. Recently, an experimental IEDF study carried out in SPIDER negative ion source is reported^29^. The simulation work conducted in our study aims to provide valuable insights for researchers focused on the E$\times$B based plasma devices, especially the negative ion source community working on the development of high current sources. We present a detailed investigation of spatial and temporal evolution of EDFs for electrons and ions using 2D-3V PIC-MCC model. In addition, different parametric studies are performed to understand the effect of magnetic field on the nature of EDFs. The results of this investigation provide new insights into the sensitive nature of the IEDF under varying bias potential and magnetic field profiles, compared to the EEDF. The “Discussion” section of the paper explains the reasons behind this sensitivity.

**Figure 1:**
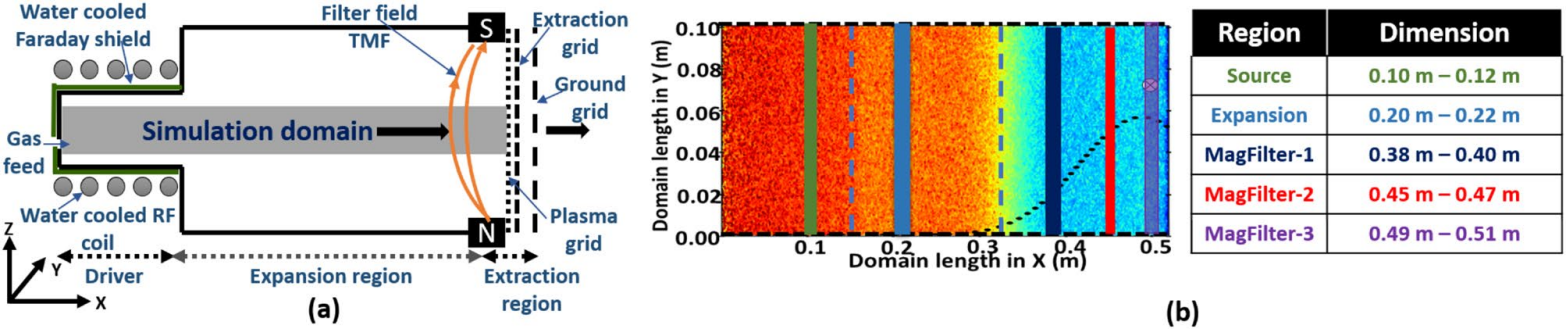
(**a**) Schematic of ROBIN ion source configuration, showing the simulation domain, different regions, TMF location, and plasma grid which can be biased independently. (**b**) The simulation domain is in the XY plane. The magnetic filter field is in the +Z direction whose Gaussian profile is shown in the black dotted line. Dashed black lines on top and bottom horizontal boundaries show periodic boundaries (no wall loss). The Blue dashed line shows the boundaries of different regions in the simulation domain. Different color lines are showing regions where EDFs are calculated. The table on the right-hand side shows the region with dimensions on X-axis.

The Rf Operated Beam source in India for Negative ion research (ROBIN), commissioned in Institute for Plasma Research (IPR), India has been considered as the reference ion source for this study^15,30,31^. Details of ROBIN are available in refs^30–33^. As shown in Fig. 1a, ROBIN has four distinct regions—(1) *driver or source region*, where plasma is created; (2) *expansion region*, (3) *transverse magnetic filter (TMF) region*, and (4) *extraction region*, from where ions start escaping out for beam extraction. In such an ion source, the magnetic filter field is in the range of 1–10 mT so that it only magnetizes electrons, and ions remain un-magnetized or weakly magnetized. The TMF profile is in Gaussian shape and perpendicular to the plasma flow from the source region to the extraction region. The plasma-facing grid (*Plasma grid*, PG) is the extreme right-hand side boundary of the extraction region which can be biased with a potential (called PG bias potential) to influence the plasma flux crossing the transverse magnetic filter. PG bias with positive potential controls the co-extracted electron current during the beam extraction. A combination of an in-homogeneous TMF field and the PG bias voltage introduces charge separation and hence relatively sharp potential gradient occurs in the magnetic filter region. This sharp potential drop is due to a double layer (DL) formation^34,35^. The characteristics of the DL strongly depend on the electron and ion velocities^35–37^. Splitting in ion velocities, a signature of DL is also reported in the TMF region^35^. In our earlier works, we have performed detailed studies on the background plasma consisting of electrons and $H_2^+$ ions in the context of negative ion sources^32,38^. In the plasma volume, the fraction of negative ions produced through volume processes is too small to influence the overall plasma dynamics^39^. However, near the ion extraction region where electron temperature is low and the surface production route is dominating, the contribution of negative ions needs to be considered with equal weightage. Given the scope of our study, which centers on plasma transport in the magnetic filter region, any effects of negative ion production through surface processes on the background plasma profiles were found to be negligible.

EDF of a species (electrons or ions $H_2^+$), defined as the number of species per unit energy range, is calculated using the Eq. (1), considering all degrees of freedom associated with the species^40,41^:

1 $$\begin{aligned} f_\varepsilon = \int _{\varepsilon _0}^{\varepsilon _f} P(\varepsilon )d\varepsilon \approx \sum _{\varepsilon _0 \le \varepsilon \le \varepsilon _f} P(\varepsilon )d\varepsilon \end{aligned}$$

where $f_\varepsilon$: non-normalized energy distribution function, $P(\varepsilon )d\varepsilon$ = $\frac{n(\varepsilon )d\varepsilon }{N}$= probability of finding particles with energy $\varepsilon$ within bin of bin size $d\varepsilon$, $n(\varepsilon )d\varepsilon$: number of species $n(\varepsilon )$ in the energy range $d\varepsilon$ in a given region, N: total number of particles (species), $\varepsilon _0$: initial energy, and $\varepsilon _f$: final energy. In our study, $\varepsilon _0$ = 0 eV, and $\varepsilon _f$ = 100 eV for EEDFs and 25 eV for IEDFs. EDF is normalized by dividing EDF with a maximum of all $\frac{n(\varepsilon )d\varepsilon }{N}$ in each regions (or each times) of the simulation domain in a single plot. Normalized EDF $g_{\varepsilon }$ is calculated by dividing EDF $f_\varepsilon$ by MAX (=$max\{P(\varepsilon )d\varepsilon \}$).

The simulation domain used in this study is XY 2D plane (as shown in Fig. 1b). Velocities of the particles are in 3D space. The periodic boundary condition (no wall loss) is applied to the top and bottom boundaries of the simulation domain (as shown by the dashed line in Fig. 1b). In the simulation domain, the left and right boundaries are kept at 0V (no bias voltage is applied). The voltage given to the right-hand side boundary (PG surface) near the end of the magnetic filter is known as the bias voltage. The details of the 2D-3V PIC-MCC simulation model are given in the “Methods” section.

## Results

**Figure 2:**
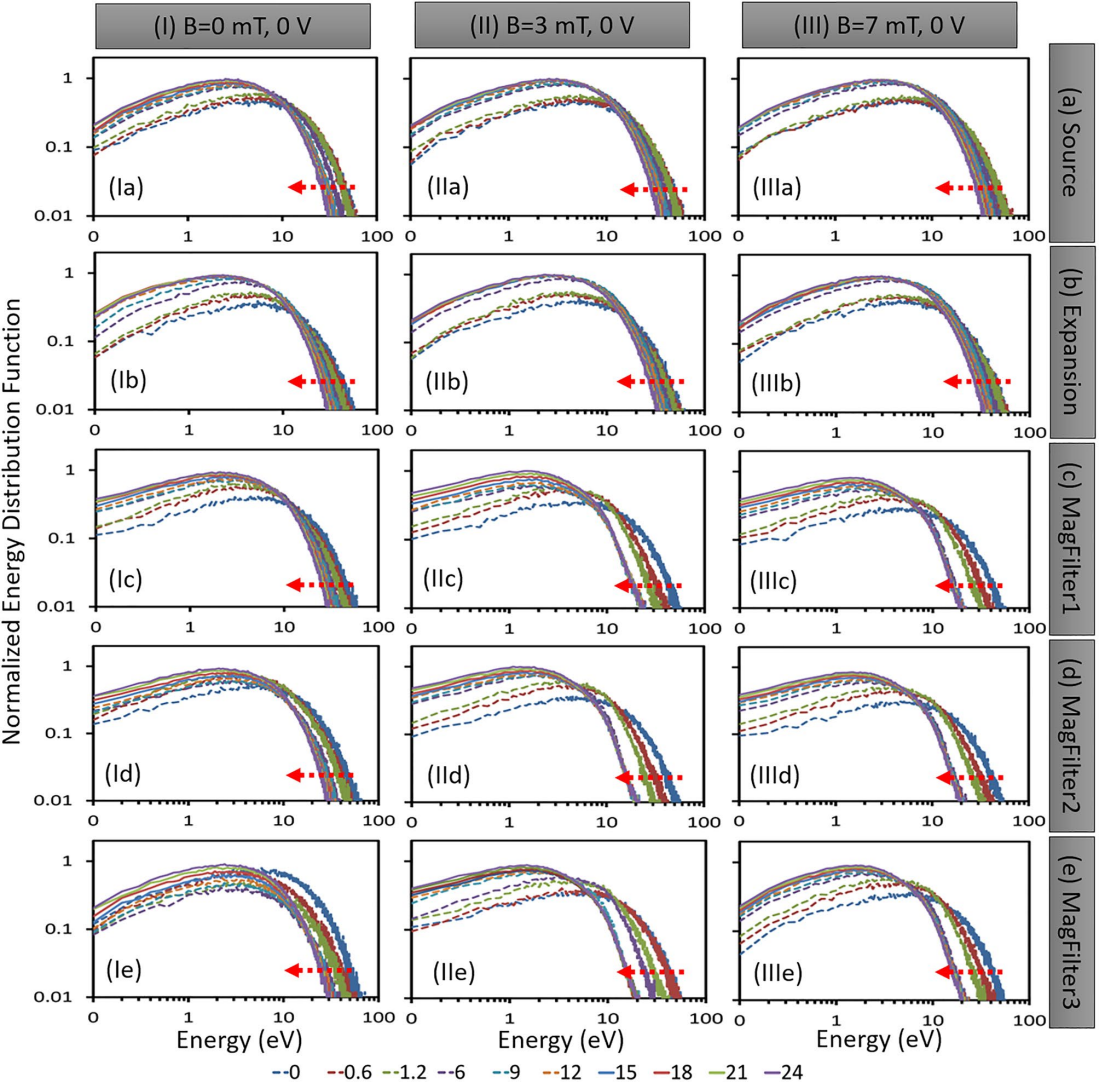
Spatio-temporal evolution of EEDF in case of three different magnetic fields with 0V bias: (I) 0 mT, (II) 3 mT, and (III) 7 mT. EEDFs are computed at different times (0 $\mu$s, 0.6 $\mu$s, 1.2 μs, 6 μs, 9 μs, 12 $\mu$s, 15 μs, 18 μs, 21 μs, and 24 $\mu$s) in five different regions of the simulation domain. Different regions are (**a**) source (0.10–0.12 m), (**b**) expansion (0.20–0.22 m), (**c**) MagFilter1 (0.38–0.40 m), (**d**) MagFilter2 (0.45–0.47 m), and (**e**) MagFilter3 (0.49–0.51 m). The arrow in each figure shows the time advancement direction.

The data obtained from our 2D-3V PIC-MCC simulations are used to calculate EDFs in different regions of the simulation domain. At the start of the simulation, velocities (EDFs) of particle species are initialized with a Maxwellian distribution corresponding to respective initial temperature considerations which later are modified or evolve self-consistently in time and space. The EDFs are modified due to various collisions, drifts, and instabilities created by the magnetic field profile and self-consistent local electric fields. Figure 1b depicts five distinct regions within the simulation domain, which are color-coded and used to calculate the energy distribution functions (EDFs) in different areas. The black dotted line in the figure represents the transverse magnetic field profile. The right-hand side table in Fig. 1b shows the dimensions of the five different regions. Figure 2 shows a complete spatio-temporal map of EEDFs in log-log scale at different times in different regions of the simulation domain (source, expansion, and three different sample zones in the magnetic filter regions as marked in Fig. 1b) for three different magnetic field configurations, without any PG bias voltage (*Case-I, No magnetic field; case-II, 3mT magnetic field, and case-III, 7mT magnetic field*).

**Figure 3:**
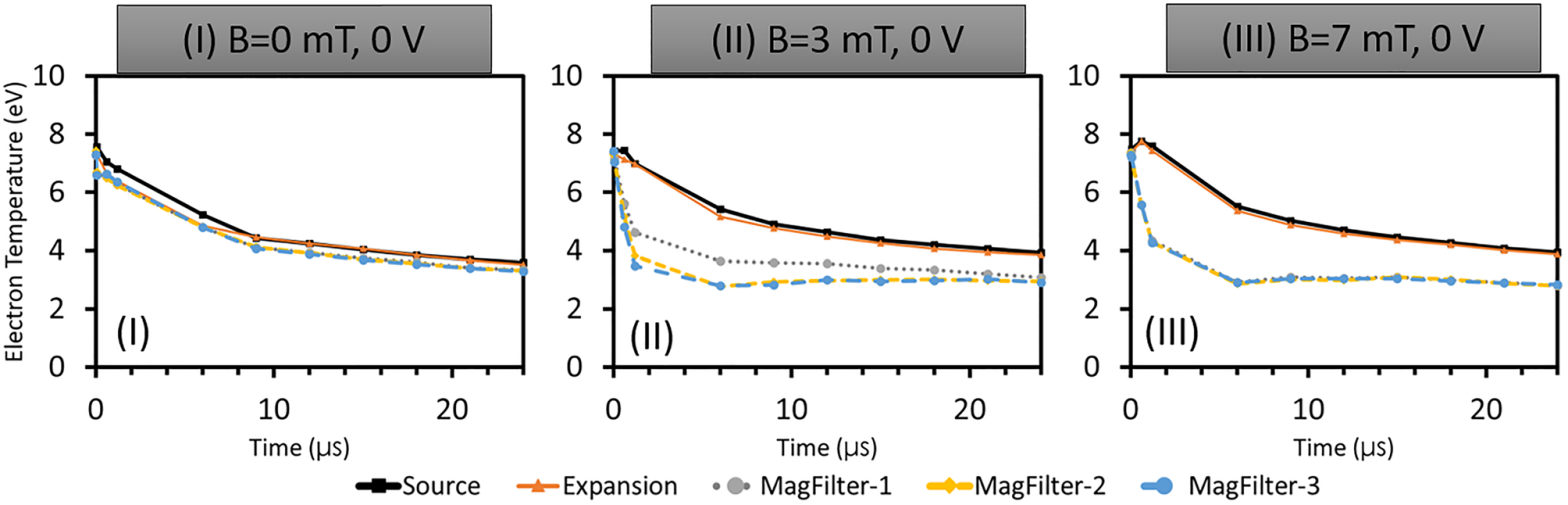
Electron temperature calculated from EEDFs for different time for different marked areas (source, expansion, MagFilter-1, MagFilter-2, and MagFilter-3) in the Fig. 1b. (**a**) 0 mT, (2) 3 mT, and (3) 7 mT. All three cases are with 0 V bias potential.

All EEDFs at different regions within the simulation domain are Maxwellian in nature, which agrees with similar kinds of low-temperature plasma experiments having magnetic filters^21,42^. The analysis of the EEDFs, shown in Fig. 2, reveals that the source region shows a broader width with a long tail due to power coupling considerations^32^, indicating the presence of more energetic electrons. As electrons travel from the source region to expansion region and then to the magnetic filter region, the width of EEDFs became narrower with a shorter tail indicating a gradual decrease in plasma temperature (nearly 5.5 eV in source and 3 eV in magnetic filter region). Additionally, the peak of each EEDF shifts towards lower energies as it moves from the source region to the magnetic filter region. EEDFs change abruptly when electrons move from expansion region to the magnetic filter region. In addition, there is a significant difference between the EEDFs obtained in the magnetic filter region^32^. This indicates that within the magnetic filter region, plasma characteristics are changed rapidly in space.

As seen in Fig. 2, generally EEDFs become narrow as time progresses in all the regions because electrons lose their energies through collisions. The stability of simulation results depends on low-frequency events linked with ion dynamics. In addition, ion has a residence time in the order of a few $\upmu$s. EEDFs become stable after 6 $\upmu$s in case-II and case-III as shown in Fig. 2. From our simulations, we find that EEDFs remain Maxwellian due to high collision frequencies^32^, and the nature of EEDFs is not sensitive to the magnetic field values; however, the width of the distributions go down with time. The results show that the width reduction is more pronounced at higher magnetic fields, suggesting that the plasma is colder under these conditions. We have estimated the electron temperature using EEDFs. After 10 $\upmu$s, electron temperature become stable as seen in Fig. 3. Figure 3 clearly shows low temperature ($\sim$ 3 eV) in the magnetic filter region (MF-2 and MF-3).

**Figure 4:**
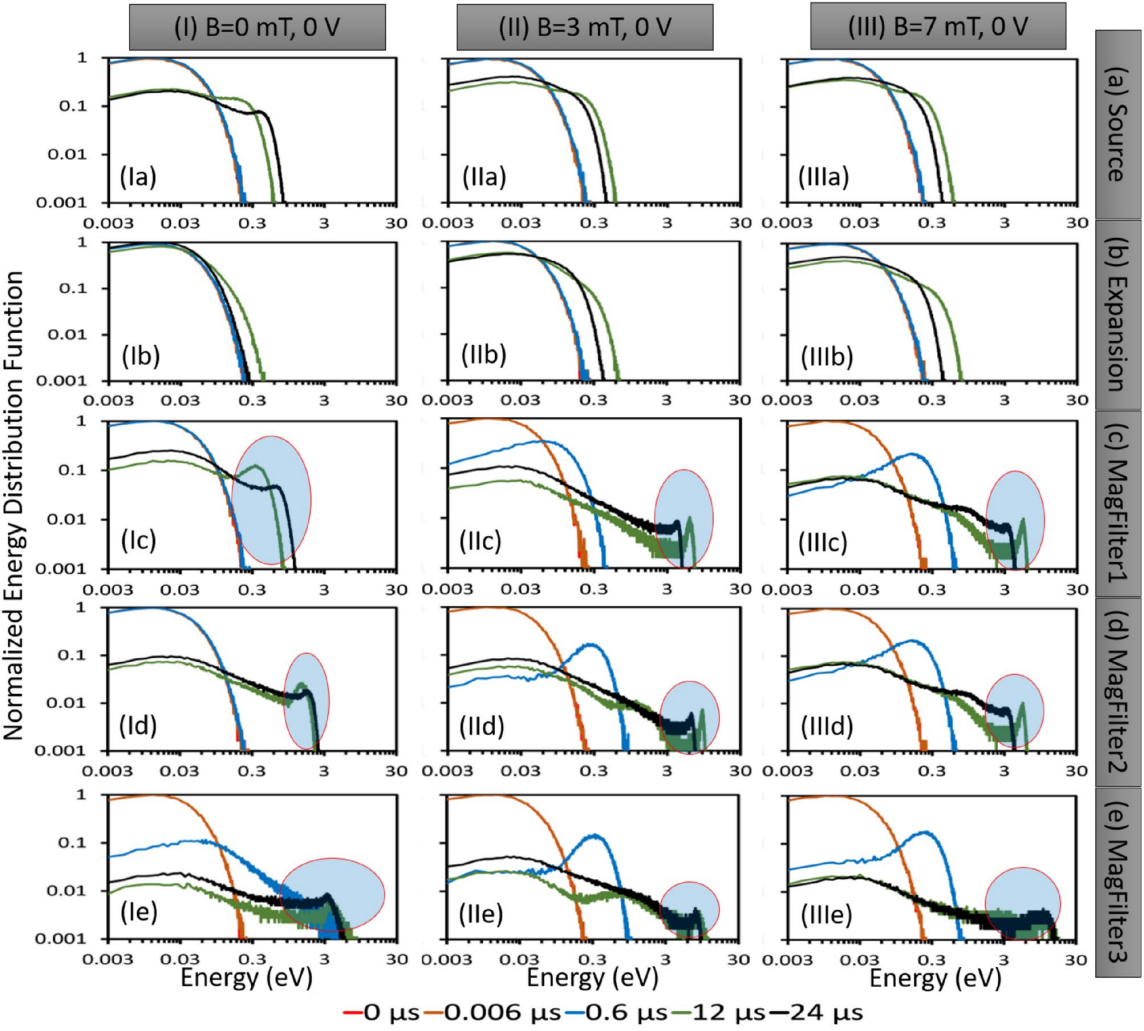
Spatio-temporal evolution IEDF in case of three different magnetic fields without PG bias (0V): (I) 0 mT, (II) 3 mT, and (III) 7 mT. IEDFs are computed at different times (0 $\upmu$s, 0.006 $\upmu$s, 0.6 $\upmu$s, 12 $\upmu$s, and 24 $\upmu$s) in five different regions of the simulation domain. Different regions are (**a**) source (0.10–0.12 m), (**b**) expansion (0.20–0.22 m), (**c**) MagFilter1 (0.38–0.40 m), (**d**) MagFilter2 (0.45–0.47 m), and (**e**) MagFilter3 (0.49–0.51 m). The blue shaded area within red circle highlights second-peak due to accelerated ions.

After analyzing the EEDFs for different configurations, the next step would be to examine the corresponding temporal evolution of ion EDFs (IEDFs). This information can provide further insight into ion transport in similar ion source configurations. The temporal evolution of IEDFs for similar configurations is presented in Fig. 4. The PIC simulation utilized in this study assumed a uniform Maxwellian IEDF with a low ion temperature of 0.1 eV as the initial condition. This means that ion velocities are distributed uniformly across the simulation domain. As it evolves with time, we observe that IEDF becomes non-Maxwellian, and a secondary peak appears near the high energy side in presence of the magnetic field. IEDFs are nearly Maxwellian in source and expansion regions as shown in Fig. 4Ia–b,IIa–b and IIIa-b. Whatever relatively small bi-Maxwellian nature is visible, is due to the sheath-presheath electric field formed on the source backplate surface (left-hand surface in the simulation domain). But as one moves towards the extraction side (i.e. MagFilter1—0.38 to 0.40 m, MagFilter2—0.45 to 0.47 m, and MagFilter3—0.49 to 0.51 m), IEDFs become prominently non-Maxwellian, and the width of IEDFs becomes wide (i.e. ions have higher energies). This nature of IEDFs is opposite to the trend seen in EEDFs shown in Fig. 2. IEDFs are the widest in the MagFilter3 region (near the extraction boundary). The increase in ion energy is due to the influence of the resultant electric field comprised of (a) sheath-presheath electric field on the extreme right-hand side surface of the simulation domain (PG surface), (b) electric field due to applied bias potential on that PG surface, and (c) electric field generated by the DL in the TMF zone^35^. The resultant electric field influences the velocity of the ions in these zones. TMF field significantly changes the IEDFs as shown in Fig. 4IIc-e and Fig. 4IIIc-e. There are significant differences between cases without a magnetic field (case-I) and with a magnetic field (case-II for 3mT maximum magnetic field and case-III for 7mT maximum magnetic field). IEDFs become broader and non-Maxwellian in case-IIc (Fig. 4IIc) and case-IIIc (Fig. 4IIIc) than case-Ic in Fig. 4Ic. These changes are due to the DL formation in the presence of the magnetic filter field^11^. Like EEDFs, the IEDFs also reach a stable state after 6 $\upmu$s. The figures suggest that the Non-Maxwellian IEDFs consist of two superimposed distributions: one with cold bulk ions that are randomly distributed, and the other with accelerated ions caused by the resultant electric field generated by either presheath-sheath or double layer.

**Figure 5:**
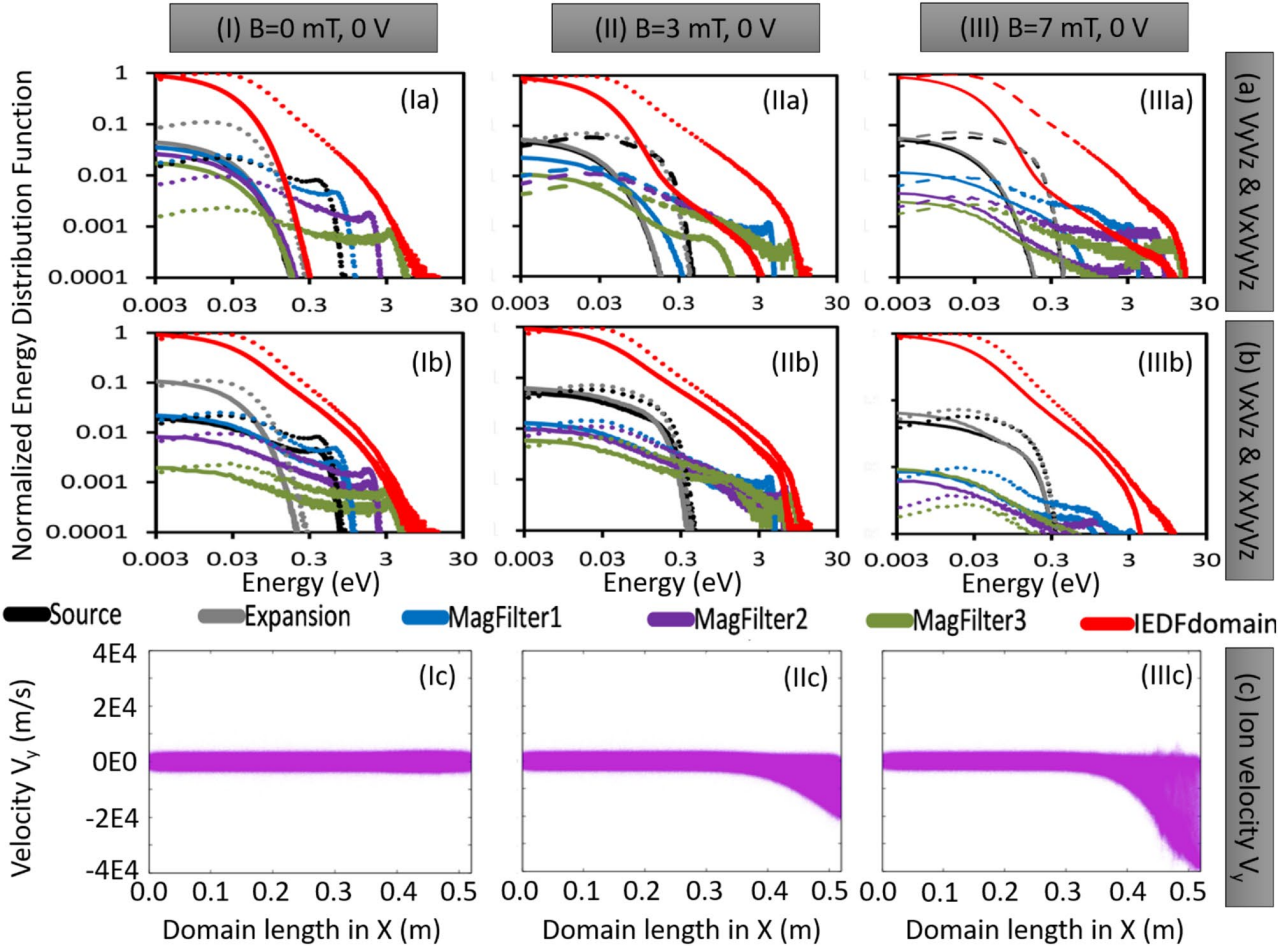
Component wise Ion Energy Distribution Function (IEDF). Dashed line shows IEDFs calculation from all three velocity components ($V_x$, $V_y$, and $V_z$). Solid line shows IEDFs from two velocity components [In, (**a**) $V_y$ and $V_z$ and (**b**) $V_x$ and $V_z$]. (**c**) Ion velocity in the Y-direction in m/s is also shown.

**Figure 6:**
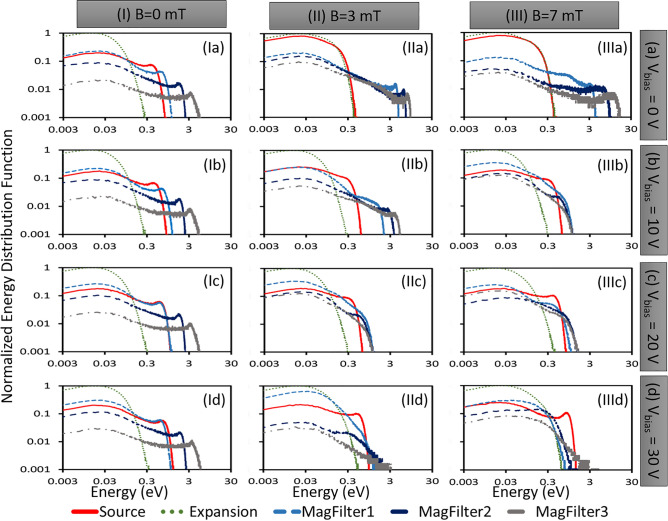
IEDFs for four different bias voltages [(**a**) 0 V, (**b**) 10 V, (**c**) 20 V, and (**d**) 30 V] and three different magnetic fields [(I) 0 mT, (II) 3 mT, and (III) 7 mT]. IEDFs calculated at 24 $\mu$s (after stability).

EDFs give information about energy components corresponding to all three velocity components ($V_x, V_y,$ and $V_z$). The information about a particular velocity component is not observed in the EDFs. However, the information about the velocity components can be captured by recalculating energies using specific velocity components. The present simulation methodology has the flexibility to identify the velocity components driven by X-axis electric fields (sheath, DL) and Y-axis drifts (E$\times$B drift, diamagnetic drift, etc.). Fig. 4 shows IEDFs calculation using all the velocity components. Fig. 5a compares two different IEDFs; first, calculated using ($V_y$ and $V_z$) to understand the influence of the drift by neglecting $V_x$ component due to axial electric field; and second, calculated using all the velocity components ($V_x, V_y$, and $V_z$). The area under IEDFs represents number density. Differences between areas under two IEDFs show the effect of the $V_x$ components on the ions (see Fig. 5a). In the absence of the magnetic field (case-1 0 mT in Fig. 5Ia), nearly 40 % of ion density accelerates in the X direction. But ion density population accelerated in the X direction decreases with increasing magnetic field (21 % in case-II (3 mT) in Fig. 5IIa and 14 % in case-III (7 mT) in Fig. 5IIIa). This indicates the contribution of cross-field drift in presence of the TMF field influences the IEDF significantly.

## Discussion

In our simulations, we aimed to replicate real-world experimental scenarios as closely as possible. The plasma is allowed to evolve over time while being subjected to heating in the *driver* region with a MHz frequency, mirroring the conditions observed in the actual experiments^31^. Throughout the simulations, we closely monitored the evolution of plasma profiles under these specific conditions. To ensure the credibility of our results, we carefully benchmarked the simulation outcomes with the corresponding experimental data^32^. Notably, the drifts and instabilities were consistently observed in our simulations as well^11,32^. This alignment between the experimental and simulated observations confirms the physical relevance and accuracy of our kinetic simulations. It is essential to recognize that kinetic simulations necessitate certain initial assumptions to initiate the plasma evolution over time. While we acknowledge this fact, we want to emphasize that these assumptions are rooted in the experimental parameters and conditions, making them a valid starting point for our investigation. As shown in our earlier work^32^, plasma parameters such as electron temperature and plasma density starts converging after 10 $\upmu$s, and if we take different initial profiles, EEDFs and IEDFs evolve differently till 10 $\upmu$s, however they show a similar trend after the convergence of plasma parameters.

In the results section, we presented the evolution of EDFs for electrons and ions at various locations in the plasma volume and under different operational scenarios of the ion source plasma. As expected, the EEDFs remained Maxwellian and the energy content and density decreased as one moved from the source region to the extraction region. However, the behavior of IEDFs is different from that of EEDFs. When a 0 V bias voltage (without PG bias) is applied at the extraction boundary, the EEDF remains relatively unchanged over time (Fig. 2), whereas the IEDF changes rapidly (Fig. 4). Additionally, Fig. 4 shows that the IEDFs have long tails with a characteristic “second peak” observed near 10 eV (shown by the blue shaded area in the red circle).

In all the non-Maxwellian IEDF plots (Figs 4, 5a,b, 6), the characteristic *second peak* is observed, which represents a beam like ionic population fraction having higher velocity or energy. Considering the case of 0 mT and 0 V bias, the *second peak* of IEDFs shifts from 1 to 3 eV range when the location shifts from MagFilter1 to MagFilter2 region. The *second peak* moves to 10 eV when the position reaches near the extraction region (MagFilter3) close to the right-hand side bias surface which is kept at 0 V. Ions got accelerated due to the sharp potential gradients near the boundaries due to the presheath-sheath. In the case of the magnetic field (case-II and case-III), the *second peak* of IEDF shifts from 8 to 11 eV when the location changes in the magnetic filter region (from MagFilter1 to MagFilter3).

In the 0 V bias case, the PG bias voltage is lower than the plasma potential, and an ion sheath is expected to be formed on the PG surface. Electric field due to presheath-sheath region accelerates the ions^43^. However, it is important to find out what will happen in the absence of an ion sheath. The condition can be achieved by increasing bias potential to compensate the floating sheath potential. Consequently, the sheath width and the ion acceleration within the sheath will decrease. Therefore, a set of simulations with four different bias voltages are considered, (a) 0 V and (b) 10 V (bias voltage less than plasma potential, condition for ion sheath), (c) 20 V (nearly same as plasma potential), and (d) 30 V (bias voltage more than plasma potential, condition for electron sheath). Fig. 6 shows IEDFs for four different bias voltages (0 V, 10 V, 20 V, and 30 V) and three different magnetic fields (0 mT, 3 mT, and 7 mT). In those cases with the magnetic field (case-II and case-III), IEDFs become narrow as bias voltage increases from 0 to 20 V (Fig. 6IIa–c,IIa–c) and then become wider from 20 to 30 V (Fig. 6IIc–d, IIIc–d). Without a magnetic field (0 mT cases), IEDFs do not change much with changing bias voltage (Fig. 6I). As the bias voltage increases in the case of absence of magnetic field (case-I), the plasma potential also increases^13^. However, despite this increase in plasma potential, the shape of the ion energy distribution function (IEDF) remains similar for different bias voltages. This is because the potential difference between the plasma potential and bias voltage remains nearly same, resulting in a similar IEDF shape across different bias voltages^13,35^. Contrary to the conventional perception, our simulations show that IEDFs gradually become non-Maxwellian and are very sensitive to the magnitude of magnetic filter and applied bias voltage on the boundary surface whereas, EEDFs remain nearly Maxwellian with a gradual reduction in temperature.

To understand the IEDF behaviors, the force balance terms are equated from the simulated electric field, applied magnetic field, and by following different velocity components. The present 2D-3V PIC MCC simulation can generate plasma potential profile self-consistently^11,32,35,38,44,45^. Therefore, a corresponding electric field can be easily estimated from the simulation which can be used to study the electrical forces acting on the ions specifically to explain the observed IEDF characteristics. The non-Maxwellian nature of IEDFs can be understood by considering different force terms^9^ considering electric field E, magnetic field B, the force due to pressure gradient $\bigtriangledown$P, and the viscosity term ($mn\nu v$) due to the collisions.

2 $$\begin{aligned} mn\frac{dv}{dt}=\pm en(E+v\times B)-\bigtriangledown P-mn\nu v \end{aligned}$$

where, m-mass of the particle (electron or ion), n-plasma density assuming quasi-neutral plasma, e-electronic charge, *v*-average particle velocity, $\nu$-collision frequency. The right-hand side of the equation (2), has four different forces which are: (1) Force due to the electric field $F_E = \pm eE$. In the case of ions, the force due to the electric field is directed toward the surface, but in the case of electrons, it is the opposite, towards the volume. The force is on X-axis. (2) Force due to the magnetic field B is $F_B = \pm e(v\times B)$, in Y-direction. This term is useful to explain case-II and case-III, IEDF data where 3mT and 7mT magnetic filter field is applied externally. (3) Force due to the pressure gradient $F_{PG} = - \frac{\bigtriangledown P}{n}$, towards the wall along X-axis. (4) Force due to collision (viscosity) $F_{colli} = - mv\nu$; along X-axis, opposite to the particular motion direction.

**Figure 7:**
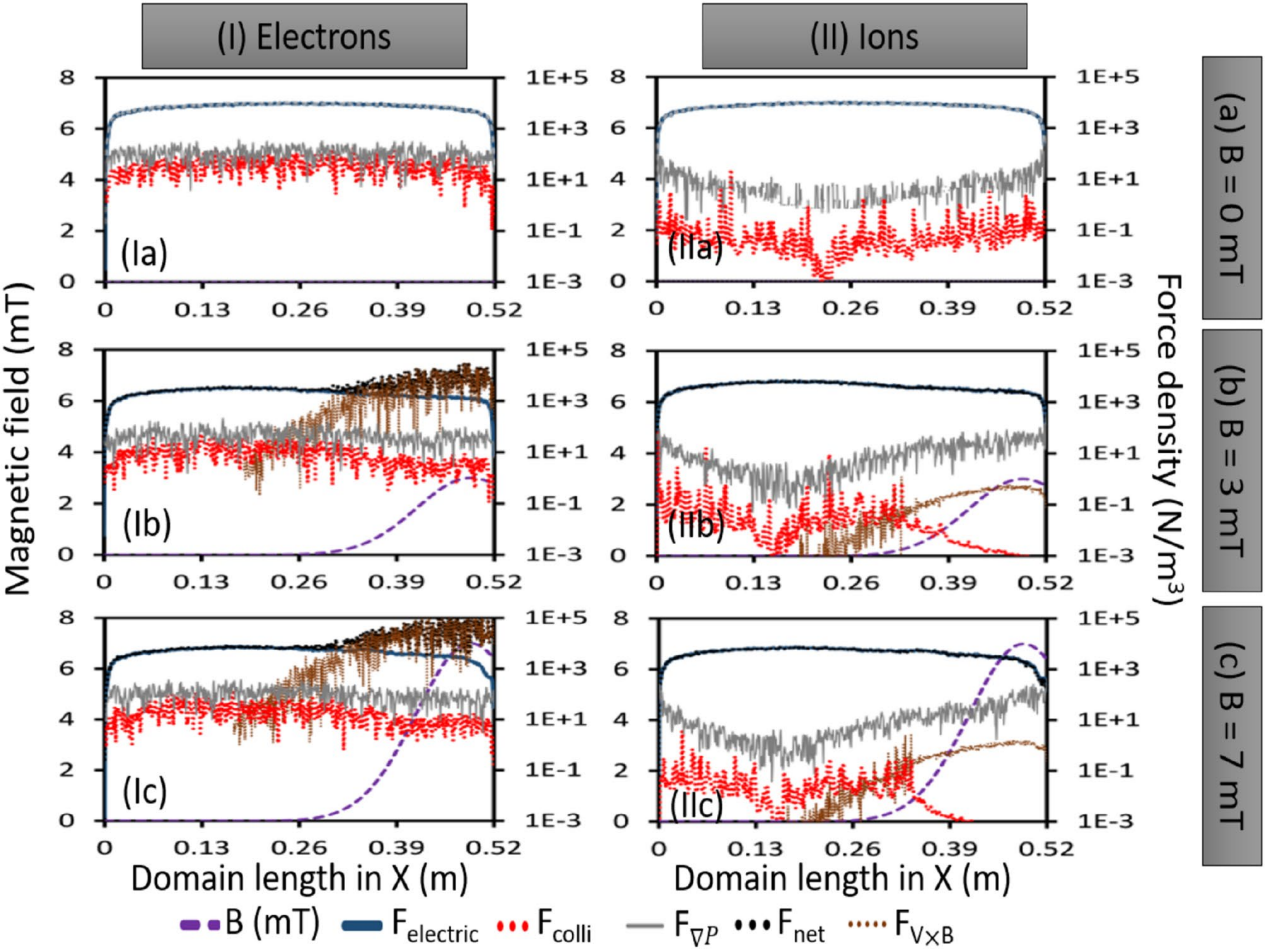
Different force density components of equation (2) in X-direction for (I) electrons and (II) ions. Three different cases of a magnetic field are considered: (**a**) 0 mT, (**b**) 3 mT, and (**c**) 7 mT. Magnetic fields is given by purple dashed line. $F_{colli}$: force density due to collision shown by red dotted line, $F_{electric}$: force density due to electric field given by blue thick solid line, $F_{\bigtriangledown P}$: force density due to pressure gradient shown by grey thin solid line, $F_{V\times B}$: force density due to $V\times B$ given by brown dotted line, $F_{net}$ : net force density shown by black dotted line..

Individual force density values (*force divided by local plasma density*) are given in Fig. 7 for 0 V bias potential. The net force on ions (black dotted line) is primarily dominated by the force due to the electric field generated from the plasma potential gradient (blue thick solid line) (Fig. 7IIa–c). On the other hand, the net force on the magnetized electrons (black dotted line) is dominated by the force due to the magnetic field (brown dotted line)(Fig. 7Ib,c).

IEDFs considering different velocity components, shown in Fig. 5b give information about the effect of the $V_y$ velocity component on IEDFs. Figure 5b presents a comparison between two IEDFs. The first IEDF is calculated considering two velocity components ($V_x$ and $V_z$), neglecting the influence of the $V_y$ component. The second IEDF is calculated taking into account all three velocity components ($V_x, V_y$, and $V_z$). Differences between areas under two IEDFs show the effect of the $V_y$ components on the ions (see Fig. 5b). In the absence of the magnetic field (case-I with 0 mT), the $V_y$ component does not affect the ion transport (Fig. 5Ib). In the case of the magnetic field (figures 5IIb,IIIb), $V_y$ component of the ions is due to the drifts in one particular direction caused by the magnetic field and its gradients and modifies $\sim$ 3–5 % of the ion transport and responsible for plasma asymmetry. Such asymmetry is observed in the ROBIN experiment and explained therein^15^.

Electron and ion velocities in the form of phase space plots helps to understand, why EDFs are Maxwellian for electrons and Non-Maxwellian for ions. In Fig. 5c, (x,$V_y$) plots are drawn for three magnetic cases without any bias voltage (0 V bias case). It clearly shows that with an increase in the transverse magnetic field (in Z-direction), the $V_y$ component (in -ve Y direction) of the ions increases (Fig. 5IIc,IIIc). This observation is attributed to the drift primarily caused by the magnetic field and its gradient. The increase in velocity of the ions in the Y-direction explains the appearance of “*second peak*” in the Fig. 5a where $V_x$ (velocity due to electric field component) is not considered. The position of “*second peak*” shifts towards the right, higher energy side when $V_x$ is also included. $V_x$ is higher in magnitude due to the electric field in the X-direction created by the presheath-sheath and the double-layer (in the case of the magnetic field).

In the source and expansion regions, IEDFs are nearly Maxwellian or weakly non-Maxwellian because the influence of magnetic field is not present but that of sheath-presheath electric fields from the source back side boundary and the right-hand side boundary of the expansion region are present. The influence of the sheath-presheath electric field is more in the source region compared to that in the expansion region due to their locations concerning the boundary surface. The impact of that electric field is visible in the ion phase-space plots as given in our previous work^35^ and also in Figs. 4 and 5a,b. However, in the magnetic field regions, IEDFs become non-Maxwellian. The additional velocity (energy) is gained due to the acceleration created by the double layers (in the X-direction) and by the drift (in the Y-direction) in the magnetic field region. As a consequence, the splitting of ion velocities is observed in some specific cases due to the accelerated velocities^35^. Bias voltage strongly affects the IEDFs by influencing the presheath-sheath electric field. IEDF tends to become Maxwellian as the bias potential is raised and goes near the plasma potential to modify the sheath effect. In the future, investigations on low-temperature plasma experiments with a similar setup having IEDF diagnostics would be interesting to understand the plasma transport across the magnetic field.

Since electrons are magnetized, it is necessary to understand how electrons behave when the sheath electric field is getting manipulated by biasing the right-hand side boundary. The phase space (x, $V_x$) of electrons for different source configurations and bias conditions are given in Ref.^35^. EEDFs in Fig. 2 become narrow with time, in the case of the magnetic filter field, due to electron trapping, losing energies within the TMF zone because of collisions and eventually, low energy filtration effect takes place. Due to magnetized conditions (case-II and case-III) and high collision frequencies electrons lose energy more compared to that of ions as shown by a narrow band in the TMF zone in the electron phase space plots^35^.

## Methods

**Table 1:** Typical Parameters used in the 2D-3V PIC MCC model.

Parameters	Value	Parameter	Value
Gas	$H_2$	Species	Electrons, $H_2^+$ ions
Gas Pressure	0.6 Pa	Plasma density	$0.2 \times 10^{14} m^{-3}$
Electron temperature	10 eV	Ion temperature	0.026 eV
Debye length	$5.26 \times 10^{-3} m$	Electron Larmonr radius	$1.08 \times 10^{-3} m$
Electron-neutral collisional mean free path	0.046 m	Ion-neutral collisional mean free path	0.69 m
Electron cyclotron frequency	$1.96 \times 10^8$ Hz	Ion cyclotron frequency	$5.34 \times 10^4$ Hz
Electron-neutral collision frequency	$2.88 \times 10^7$ Hz	Electron-ion collision frequency	34 Hz
Electron plasma frequency	$4.02 \times 10^7$ Hz	Grid size in X and Y	800 in X, 155 in Y
Time step	$0.12 \times 10^{-9}$ s	$\Delta X$ and $\Delta Y$	6.5 $\times 10^{-4}$ m

Detailed computational implementation of the 2D-3V PIC-MCC model used in this study can be found in^32,38,44^. As an initial condition, particles are loaded uniformly all over the simulation domain and velocities are initialized with the Maxwellian distribution. Particle positions and velocities are updated in the Lagrangian mesh-free grid as simulation progresses. Charge density on Eulerian grid points is calculated from particle positions. Particles to grid interpolation (*mover*) is performed using first-order weighing scheme^38,44^. The potential is calculated from charge densities using PARDISO Poisson solver^46^. Electric field is computed using gradient of the potential. Lorentz’s force is updated using new electric field and static applied magnetic field. Using Newton’s equation, the particle’s position and velocities are updated. Boris method is applied to calculate $\vec {V} \times \vec {B}$. Grid-to-particle interpolation (charge deposition) is calculated using a first-order weighing scheme. Monte-Carlo collision scheme is used to take into account sixteen types of collisional phenomena describing the important hydrogen chemistry (ionization, elastic, and inelastic collisions)^45^. Self-consistent electron heating is applied in the *driver* region of the domain (red area in Fig. 1b). Randomly selected electrons in the *driver* region are heated continuously at RF heating frequency^32^. Electrons transfer energies through collisions and plasma sustains. The other relevant typical parameters used in the study which can influence the simulation are shown in Table 1. We have used an in-house hybrid parallel 2D-3V PIC-MCC code for accelerated simulations^44^.

## Data Availability

The datasets generated during and/or analysed during the current study are available from the corresponding author on reasonable request.
